# Involvement of the Efflux Pumps in Chloramphenicol Selected Strains of *Burkholderia thailandensis*: Proteomic and Mechanistic Evidence

**DOI:** 10.1371/journal.pone.0016892

**Published:** 2011-02-09

**Authors:** Fabrice V. Biot, Eric Valade, Eric Garnotel, Jacqueline Chevalier, Claude Villard, François M. Thibault, Dominique R. Vidal, Jean-Marie Pagès

**Affiliations:** 1 UMR-MD-1, Facultés de Médecine et de Pharmacie, Université de la Méditerranée, IFR88, Marseille, France; 2 Institut de Recherche Biomédicale des Armées/CRSSA/Unité de Bactériologie/UMR-MD-1, La Tronche, France; 3 Hôpital d'Instruction des Armées Laveran, Marseille, France; 4 Plate-forme Protéomique Timone Map, INSERM UMR 911 CRO2, Faculté de Pharmacie/Université de la Méditerranée, Marseille, France; Duke-National University of Singapore Graduate Medical School, Singapore

## Abstract

*Burkholderia* is a bacterial genus comprising several pathogenic species, including two species highly pathogenic for humans, *B. pseudomallei* and *B. mallei*. *B. thailandensis* is a weakly pathogenic species closely related to both *B. pseudomallei* and *B. mallei*. It is used as a study model. These bacteria are able to exhibit multiple resistance mechanisms towards various families of antibiotics. By sequentially plating *B. thailandensis* wild type strains on chloramphenicol we obtained several resistant variants. This chloramphenicol-induced resistance was associated with resistance against structurally unrelated antibiotics including quinolones and tetracyclines. We functionally and proteomically demonstrate that this multidrug resistance phenotype, identified in chloramphenicol-resistant variants, is associated with the overexpression of two different efflux pumps. These efflux pumps are able to expel antibiotics from several families, including chloramphenicol, quinolones, tetracyclines, trimethoprim and some β-lactams, and present a partial susceptibility to efflux pump inhibitors. It is thus possible that *Burkholderia* species can develop such adaptive resistance mechanisms in response to antibiotic pressure resulting in emergence of multidrug resistant strains. Antibiotics known to easily induce overexpression of these efflux pumps should be used with discernment in the treatment of *Burkholderia* infections.

## Introduction

*Burkholderia thailandensis* is an environmental bacterial species which is considered to be an opportunist pathogen [1]. There are only few documented cases of infectious diseases in human associated with this Gram-negative bacterium and they generally involve immunocompromized patients already suffering from other diseases [2], for example cystic fibrosis (unpublished data). The genome of *B. thailandensis* shows extensive similarities with the genomes of *B. pseudomallei*, the causative agent of melioidosis and of *B. mallei*, the etiological agent of glanders [3], [4]. The treatments of these infections are both cumbersome and prolonged, and involve the administration of multiple antibiotics [5], [6]. These bacterial species express several resistance mechanisms leading to a decreased sensitivity to many antibiotics [7], [8]. Furthermore, there are cases of emergence of resistant isolates during treatment in patient with relapse with the same strain that it can generate therapeutic failures [9], [10]. The best described resistance mechanisms in this bacterial genus are β-lactamases and Multi Drug Resistance (MDR) efflux pumps [11]. *B. thailandensis*, biochemically and genetically closely related to *B. pseudomallei* and to *B. mallei*, is a useful model system for studying the resistance mechanisms in *Burkholderia* spp. [4], [12]. Interestingly, the *B. thailandensis* genome contains many putative Resistance Nodulation cell Division (RND) efflux pump genes closely related to those of *B. pseudomallei* [4]. Four efflux pumps involved in the active extrusion of drugs have been identified in *B. pseudomallei*: AmrAB-OprA, BpeAB-OprB, BpeEF-OprC, BpeGH-OprD [13], [14], [15], [16], [17], [18], although, only the first three of these have been demonstrated to be associated with antibiotic resistance. There are also many additional putative RND efflux pumps in this species, but which have not been characterized in resistant strains [4], [19]. However, none of efflux pump has been yet described in *B. thailandensis*.

The aim of this work was to investigate the adaptive response of *B. thailandensis* in medium containing chloramphenicol and to characterize the expression of the corresponding resistance mechanisms. We used a proteomic method to assess the involvement of RND efflux pumps in the antibiotic resistances expressed by *B. thailandensis*. Chloramphenicol was chosen because we have previously demonstrated that this antibiotic is able to select *in vitro* resistant strains of Gram-negative bacteria that overproduce the AcrAB pump, a major efflux pump in clinical isolates of *Enterobacteriaceae* [20], [21]. Furthermore, chloramphenicol has been used for a long time as first intention treatment and can be still used in particular cases of melioidosis [5], [8]. To investigate chloramphenicol as an activator of the multidrug resistance phenotype in *B. thailandensis*, as previously described in *Escherichia coli* and *Enterobacter aerogenes* [22], [23], resistant variants were obtained by cultivating a susceptible strain of *B. thailandensis* in the presence of increasing chloramphenicol concentrations.

The resulting variants exhibited decreased susceptibility not only to chloramphenicol but also to other chemically unrelated antibiotics. We studied the genetic and proteomic characteristics of these strains to assess the involvement of efflux pumps in the multidrug resistance.

## Results

### Antibiotic susceptibility of *B. thailandensis* variants

*B. thailandensis* strains ATCC 700388 and PHLSE082 were grown in the presence of chloramphenicol (4–128 µg/mL) and successive chloramphenicol-resistant derivatives were isolated. We kept only four derivatives for each strain: 64CM16, 64CM32, 64CM64 and 64CM128 from the wild-type strain ATCC 700388, which were respectively grown at a concentration of 16, 32, 64 and 128 µg/mL; and 132CM16, 132CM32, 132CM64 and 132CM128 from the wild-type strain PHLSE082. The MICs of various antibiotics for these strains were determined and compared with those for the parental susceptible strains (Table 1).

**Table 1:** Antibiotic susceptibility of the *B. thailandensis* strains.

*B. thailandensis* strains	MIC (µg/mL)															
	CM	NAL	NFX	CIP	FOX	CAZ	OXA	CLO	PIP	IMI	ERY	TC	DC	TP	TS	POL B
**ATCC 700388**	8/16	32	16	4	1024	4	128/256	256	4/8	0.19	256	6	2	8	0.75	>2048
**64CM16**	128/256	256	64/128	16	>4096	2	1024	1024	8/16	0.19	512	12	8	>32	1	>2048
**64CM32**	256	256	64/128	16	>4096	4	1024	1024	8	0.19	512	12	12	>32	1.5	>2048
**64CM64**	256	256/512	64/128	16	>4096	2	1024	2048	8	0.25	512	24	24	>32	>32	>2048
**64CM128**	512	512	128	32	>4096	2	1024	1024	8	0.125	512	12	12	>32	4	>2048

Antimicrobial agent abbreviations: CM, chloramphenicol; NAL, nalidixic acid; NFX, norfloxacin; CIP, ciprofloxacin; FOX, cefoxitin; CAZ, ceftazidime; OXA, oxacillin; CLO, cloxacillin; PIP, piperacillin; IMI, imipenem; ERY, erythromycin; TC, tetracycline; DC, doxycycline; TP, trimethoprim; TS, trimethoprim/sulfamethoxazole; POL B, polymyxin B. Values are means of three independent determinations.

The resistant variants from the two wild-type strains ATCC 700388 and PHLSE082 exhibited significant decreases of susceptibility not only to the chloramphenicol but to other structurally-unrelated antibiotics including quinolones, fluoroquinolones, trimethoprim, trimethoprim-sulfamethoxazole, and the β-lactams oxacillin and cloxacillin, and doxycycline (Table 1 and data not shown). By contrast, the MICs for erythromycin, ceftazidime, imipenem and piperacillin for the variants did not differ substantially from those of the parental strains. Specific mechanisms, such as target mutation or enzymatic modification of drugs, can only confer cross resistance within single antibiotic families. The decrease of susceptibility to antibiotics of different classes —chloramphenicol, quinolones, cyclines, trimethoprim, some β-lactams— therefore suggests that the resistant strains express a non-specific resistance mechanism, such as membrane modification or efflux pump overexpression. The MICs of imipenem, ceftazidime or piperacillin are not affected in the chloramphenicol resistant strains. These molecules use the porin way to penetrate the outer membrane [24] and this suggests that porin alterations are not involved in the induced multidrug resistance. In order to assess a possible modification of the outer leaflet constituted by the lipopolysaccharide (LPS), we tested various EDTA concentrations (1 and 2 mmol/L) during MIC determination. It has been previously observed that the addition of chelators such as EDTA is able to induce decrease of MICs [25], [26]. The susceptibilities to chloramphenicol, polymyxin B and nalidixic acid were not increased in the presence of EDTA in the chloramphenicol resistant strains (data not shown). This suggests that, in the various chloramphenicol resistant strains, the modification of the bacterial susceptibility to tested antibiotic molecules cannot be associated with a LPS modification including drastic changes in structure and charges [27].

### Effect of efflux pump inhibitors on antibiotic susceptibility

To assess the involvement of an active efflux of antibiotics, we compared the MICs for chloramphenicol-resistant strains in the presence and absence of two well-characterized efflux pump inhibitors (EPI), PAβN and verapamil, and also of the quinazoline derivative 1190 [28]. For all strains, MICs of PAβN and quinazoline derivative 1190 were greater than 2048 µg/mL and MICs of verapamil were greater than or equal to 512 µg/mL (data not shown). We tested whether various different sub-inhibitory concentrations of the three EPIs restored antibiotic activity. The presence of PAβN partially restored the antibiotic activity of chloramphenicol, nalidixic acid and cloxacillin: at about 1/10^th^ of its MIC for efflux, PAβN reduced the MICs of these three structurally-unrelated molecules by about 2-4-fold (Table 2). Note that cloxacillin is a substrate of efflux pumps and its efflux is decreased by the presence of PAβN [28], [29]. Quinazoline derivative 1190 reduced the MICs of chloramphenicol by about 2-4-fold whereas verapamil, an inhibitor of ABC drug transporters, had no significant effect, even at high concentrations, on chloramphenicol susceptibility (data not shown).

**Table 2:** Effects of PAβN on antibiotic susceptibility of the *B. thailandensis* strains.

	MIC (µg/mL)															
	CM						NAL						CLO			
	PAβN (µg/mL)						PAβN (µg/mL)						PAβN (µg/mL)			
**strains**	0	20	50	80	100	200	0	20	50	80	100	200	0	50	100	200
**ATCC 700388**	16	16	16	16	8	8	32	32	32	32	16	16	256	64	64	32
**64CM32**	256	256	256	128	128	64	256	256	256	128	128	64	1024	256	128	64
**64CM128**	512	512	512	512	512	128	512	512	512	256	128	64	1024	512	512	256

Abbreviations: PAβN, phenylalanine-arginine β-naphthylamide; CM, chloramphenicol; NAL, nalidixic acid; CLO, cloxacillin.

### SDS-PAGE analysis of membrane fractions from the various *B. thailandensis* strains

**Figure 1:**
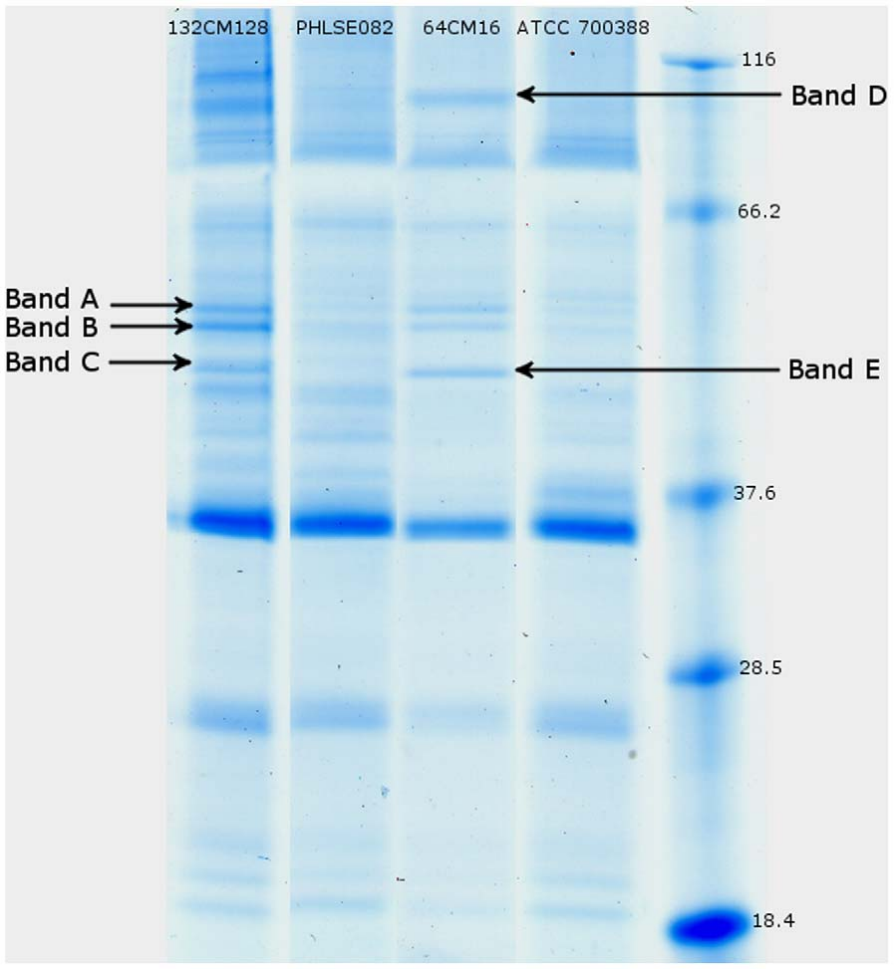
Analyses of the detergent-insoluble membrane proteins of chloramphenicol-resistant variants. SDS-PAGE analysis was performed on ATCC700388 and PHLSE082, two wild-type strains of *B. thailandensis*, and on 64CM16 and 132CM128, their respectively chloramphenicol resistant derivative strains. Proteins were stained with Coomassie blue. The variants presented different additional bands at around 51 kDa (band A), 48 kDa (band B), 43 kDa (bands C and band E) and at 95 kDa (band D). Molecular weight standards are indicated in kilodaltons.

The proteins present in the detergent-insoluble membrane fractions obtained from the various resistant and parental strains were analyzed by SDS-polyacrylamide gel electrophoresis (Figure 1). Protein staining revealed differences in the intensities of some bands between the variants and the wild-type strains. Proteins migrating at around 51 kDa (band A), 48 kDa (band B), 43 kDa (band C and band E) and at 95 kDa (band D) seemed to be more abundant in the membrane fractions derived from the resistant variants than those from the parental strains. These differences in band intensities were reproducibly observed in several independent SDS-PAGE analyses. The corresponding proteins, excised from gels, were identified by mass spectrometry. Due to the strong interactions between the components of efflux pumps (outer membrane channel, periplasmic adaptator, inner pump), we observed a “co-fractionation” of the different membrane proteins during the detergent extraction.

### Identification of drug efflux pumps overproduced in the resistant variants

Various bands (A, B, C, D and E), absent or weakly expressed in the wild-type and more abundant in resistant strains, were excised from the gels; the proteins were retrieved and used for mass spectrometry determinations. The mass spectrum of band A (51 kDa) matched against the NCBR (Genbank) bank was identified as BTH_I0682 (54,799 Da) an outer membrane channel reported in *Burkholderia thailandensis* strain E264 [3]. This outer membrane channel of the RND efflux pump presented 99% identity with a putative protein named BTH_II1286. A homologous protein (99%) named OprB of these proteins has been described in *B. pseudomallei*: it corresponds to the outer membrane channel of the RND efflux pump BpeAB-OprB [13]. In *Pseudomonas aeruginosa* PA01, the homologue of BTH_I0682 is OprM (56% amino acid sequence identity) (Table 3) [30].

**Table 3:** Identification of two drug efflux pumps overproduced in the resistant variants.

Band no.	Assession no.	Protein	Mol. wt. (Da)	Protein score	% coverage	Peptide (Hits)	Homologues in*B. pseudomallei* 1106b	Homologues in*P. aeruginosa* PAO1
A	GI|257142607	BTH_I0682BTH_II1286	54,79957,702	130.24	26.51	57	OprB99% identities	OprM56% identities
B	GI|257140923	BTH_II2104	54,869	100.17	25.49	12	OprC99% identities	No homologous protein
C	GI|257140098	BTH_I0680	43,086	160.22	45.99	45	BpeA99% identities	MexA53% identities
D	GI|257140097	BTH_I0681	114,571	250.19	21.48	69	BpeB99% identities	MexB64% identities
E	GI|257140098	BTH_I0680	43,086	140.28	46.52	50	BpeA99% identities	MexA53% identities

The bands C and E (43 kDa) were identified as the same protein, another component of RND efflux pump: the Major Facilitator Protein (MFP) subunit, periplasmic adaptator or membrane fusion protein. The name of the corresponding locus is BTH_I0680 (43,086 Da). This subunit is the homologue of BpeA of *B. pseudomallei* and MexA of *P. aeruginosa* (Table 3) [30].

Band D (93 kDa) was identified as BTH_I0681 (114,571 Da) which is a putative pump homologue of BpeB of *B. pseudomallei* and MexB of *P. aeruginosa* [30].

Band B (48 kDa) was identified as BTH_II2104 (54,869 Da). This protein is the homologue of OprC in *B. pseudomallei*. OprC has been described to be the outer membrane channel of the RND efflux pump BpeEF-OprC [14]. No homologous putative protein was found in the *P. aeruginosa* PA01 genome.

These related proteins were more abundant in the membrane fraction of the resistant strain than parental strains. Similar overproduction of these membrane proteins was found in strains selected by chloramphenicol from the both wild-type strains (data not shown). Presumably, the RND efflux pumps identified as being overproduced in the chloramphenicol-resistant variants are involved in the MDR phenotype.

## Discussion

*B. thailandensis* is a Gram-negative bacterium which can cause infections in patients with other underlying disease such as immunocompromized state. Our aim was to study the *B. thailandensis* response to chloramphenicol, an antibiotic able to induce efflux in *Enterobacteriae*. Resistant variants were isolated by culture in presence of increasing chloramphenicol concentrations, as previously used for selection of antibiotic-resistant *E. aerogenes* strains [31]. By using low concentrations of antibiotics, our objective was to make the two strains, ATCC 700388 and PHLSE082, to become progressively more resistant to chloramphenicol, in a process we call “training”. We tested the MICs of various antibiotics for these variants, and found a significant decrease of susceptibility not only for the chloramphenicol, but also for other, structurally-unrelated, antibiotic families, and in particular quinolones, fluoroquinolones, tetracyclines, trimethoprim-sulfamethoxazole and some β-lactams. The MDR phenotype of these variants suggests the presence of a non-specific drug resistance mechanism, such as the decrease of antibiotic influx or the overexpression of efflux pumps, or both. Indeed, these mechanisms together constitute the bacterial mechanical barrier to antibiotics [24] and MDR mechanisms of this type have been described in other Gram-negative bacteria, notably *Enterobacteriaceae* and the more closely related *P. aeruginosa* [32].

To assess the possible involvement of efflux mechanisms in our selected MDR strains, we investigated the effect of efflux pump inhibitors on their susceptibility to antibiotics. Susceptibility to chloramphenicol, quinolones and cloxacillin was partially restored by the presence of PAβN, an inhibitor of RND efflux pumps, but not by the presence of verapamil, an inhibitor of ABC transporter [28]. A similar effect was obtained with compound 1190, a quinazoline derivative recently reported to restore partial susceptibility in *E. aerogenes*, *Klebsiella pneumoniae* and *P. aeruginosa* MDR isolates that overexpress AcrAB-TolC or MexAB-OprM efflux pumps [33]. These results suggest that the expression of an RND efflux pump, sensitive to efflux pump inhibitor, contributes to the resistance of these strains to chloramphenicol, nalidixic acid and cloxacillin. As the susceptibilities to these antibiotics are not totally restored even by using high concentrations of PAβN, we could not exclude the presence in *B. thailandensis* of additional mechanisms such as membrane modification or the expression of another efflux pump which would be unsusceptible to PAβN [13]. However it must be noted that the susceptibilities of imipenem, ceftazidime or piperacillin were not affected in the chloramphenicol resistant strains. This result suggests that the membrane permeability, regarding porins or membrane fluidity, could not be involved in the resistance level obtained in resistant strains [22]. Furthermore, we have observed no modification of the susceptibility to chloramphenicol, polymyxin B and nalidixic acid during the addition of the chelator EDTA that affects the LPS [27]. Consequently, the modification of LPS structure seems not involved in the multidrug resistance phenotype of our chloramphenicol resistant variants.

We investigated membrane proteins of the variants by 1-D SDS-PAGE combined with protein identification by trypsin digestion and MS analysis (Table 3). This analysis indicated that all the resistant variants selected by chloramphenicol overproduced at least one RND efflux pump homologous to BpeAB-OprB in *B. pseudomallei* and encoded by the operon *bth_I0680-bth_I0681-bth_I0682* on chromosome I of *B. thailandensis*. Our results are in accordance with those of Mima *et al.* who demonstrated that the homologue of BTH_I0680-BTH_I0681-BTH_I0682 in *B. pseudomallei*, BpeAB-OprB, extrudes chloramphenicol, tetracyclines and fluoroquinolones [15]. Furthermore, the antibiotics substrates of BTH_I0680-BTH_I0681-BTH_I0682 are the same of its homologue MexAB-OprM in *P. aeruginosa* [30], [34].

The presence of chloramphenicol favors the overexpression of BpeAB-OprB homologue in *B. thailandensis* as previously described for AcrAB-TolC in *E. aerogenes* [22]. It is likely that chloramphenicol is a well-recognized substrate and may act as selector for this pump expression.

However, we detected a component of another efflux pump, the outer membrane channel BTH_II2104 of the RND efflux pump BTH_II2104-BTH_II2105-BTH_II2106. This efflux pump is homologous to the BpeEF-OprC of *B. pseudomallei* 1106b [14]. BpeEF-OprC has been proposed to expel antibiotics including chloramphenicol, tetracyclines and trimethoprim [14], [35]. Overexpression of this pump may thus explain the increased MICs of chloramphenicol and trimethoprim observed for our variants. We observed overproduction of the homologue of OprC in one resistant variant (132CM128), a variant growing on the media containing the highest concentration of chloramphenicol, whereas the homologue of BpeAB-OprB was overproduced in all chloramphenicol-resistant variants. Possibly, the homologue of BpeAB-OprB is overexpressed before the homologue of BpeEF-OprC under chloramphenicol stress; the latter only being expressed under high concentrations of chloramphenicol.

We only found the outer membrane channel of this pump and not the other components homologous to BpeEF. Either SDS-PAGE analysis failed to reveal the other components of the original pump associated with BTH_II2104, or alternatively BTH_II2104 may be associated with the BpeAB homologue. Indeed, such functional combinations between components of different RND efflux pumps have been demonstrated in *P. aeruginosa* [36]. These associations between components of different RND efflux pumps may modulate the affinities of these transporters for different substrates.

We did not construct any mutants deleted in the two identified efflux pumps because it has been demonstrated that another pump could be overexpressed in order to compensate the lacking pump [37]. In our case, we can observe that at least two pumps are able to be sequentially overexpressed for maintaining the survival of the bacteria under high level concentrations of chloramphenicol.

Interestingly, susceptibility to imipenem, ceftazidime and piperacillin was preserved in the MDR variants we obtained. Possibly, the efflux pumps activated in *B. thailandensis* by chloramphenicol do not have affinity for these antibiotics. Vitkorov *et al.* [18] have recently shown that growing *B. thailandensis* and *B. pseudomallei* on media containing ceftazidime and fluoroquinolones was possible and could generate MDR with a profile similar to that we describe here in *B. thailandensis*.

In conclusion, these data demonstrated that *B. thailandensis* is able to adapt to antibiotic stress by using diverse coordinated efflux pumps. This indicates that the choice of appropriate antibiotics is essential for treatment success of *Burkholderia* infections. As a consequence, the bacterial adaptation to chloramphenicol and the resulting MDR we report lead us to conclude that it is possible that one antibiotic is able to generate a resistance to the main antibiotic families included to the oral phase of treatment of *Burkholderia* infections. It could be of interest to lead similar studies in more pathogenic species of *Burkholderia* as *B. pseudomallei* and *B. mallei* and explore the role of active efflux induced by the treatment in the cases of relapses in melioidosis and glanders.

## Materials and Methods

### Bacterial strains, growth media and selection of chloramphenicol-resistant strains

Bacteria were grown at 37°C in Luria–Bertani (LB) broth, in Trypticase soya (TS) broth or in TS agar media (Difco Laboratories, Detroit, MI, USA). *B. thailandensis* ATCC 700388 (type strain) was used as the wild-type strain. Four strains named 64CM16, 64CM32, 64CM64 and 64CM128 were sequentially obtained from the reference strain ATCC 700388 by growing on a gradient with concentration steps of 8–16, 16–32, 32–64, and 64–128 µg/mL chloramphenicol. The resulting strains, ATCC 700388, 64CM16, 64CM32, 64CM64 and 64CM128, were routinely maintained with respectively 0, 16, 32, 64 and 128 µg/mL of chloramphenicol. The same method was used for another strain of *B. thailandensis* named PHLSE082 (HPA, UK, formerly PHLS) to generate four chloramphenicol-resistant variants named, respectively, 132CM16, 132CM32, 132CM64 and 132CM128.

### Antibiotic susceptibility tests

Minimal Inhibitory Concentrations (MIC) of chloramphenicol, nalidixic acid, ciprofloxacin, norfloxacin, cefoxitin, oxacillin, cloxacillin, piperacillin, erythromycin, imipenem and polymyxin B (Merck Sharp Dohme and Chibret, Paris, France; Bristol-Myers Squibb, Paris, France; and Sigma-Aldrich, MO, USA) were measured by the broth dilution method, as previously described [38] and according to the Clinical and Laboratory Standards Institute (http://www.clsi.org – October 2010) and the Comité de l'Antibiogramme de la Société Française de Microbiologie (http://www.sfm.asso.fr/publi/general.php?pa=1 – October 2010). Approximately 10^6^ cells were used to inoculate 1 mL Mueller Hinton II broth (Becton Dickinson and Company, Sparks, MD, USA) containing twofold serial dilutions of each antibiotic. Results were read after incubation for 24 h at 37°C and are expressed as MICs in µg/mL. MICs of tetracycline, doxycycline, trimethoprim and trimethoprim-sulfamethoxazole and imipenem were measured by the Etest® method (Biomérieux). The efflux pump inhibitors, phenylalanine-arginine β-naphthylamide (PAβN), verapamil (Sigma-Aldrich, MO, USA) and a quinazoline derivative, named 1190, synthesized by our drug design laboratory, were used as previously described [39]. These efflux pumps inhibitors (PAβN, from 20 to 200 mg/L; verapamil, 50 and 100 mg/L; quinazoline derivative 1190, 162, 325 and 650 mg/L) are incorporated in the broth containing the cells so that to obtain the final given concentration. EDTA (Sigma-Aldrich, MO, USA) was used at different concentrations (1 and 2 mmol/L) in association with chloramphenicol, nalidixic acid and polymyxin B.

### Preparation of membrane fractions

Bacterial membrane fractions were prepared from 50 mL mid-exponential phase cultures. Bacteria were harvested, washed, and resuspended in 10 mL cold sodium phosphate buffer (100 mM NaH_2_PO_4_/Na_2_HPO_4_, pH 7.4) containing 1 mg/mL lysosyme. Cells were lysed by sonication (Branson Sonifier 450; 50% duty cycle; amplitude setting 20%; total time 7 min) and unbroken bacteria were removed by centrifugation (5,000 g; 20 min; 4°C). Whole membranes were recovered from the supernatant by ultracentrifugation (40,000 g; 60 min; 4°C) and incubated in 0.15% sodium N-laurylsarcosinate for 30 min at room temperature to extract the detergent-soluble material modified from the previously described procedure [22]. The insoluble membrane fractions were pelleted by centrifugation (40,000 g; 60 min; 20°C). Proteins in the pellets were resuspended in solubilisation buffer and heated for 5 min at 95°C as previously described [31].

### SDS-PAGE

Exponential growing bacteria in LB broth were pelleted and solubilised in boiling buffer at 95°C as described elsewhere [31]. Samples were loaded onto SDS-polyacrylamide gels (10% polyacrylamide, 0.1% SDS). After migration, gels were stained with Coomassie Brillant Blue R-250.

### Protein identification: peptides digestion and nano electrospray MS/MS identification

Protein bands were excised from gels and digested in the resulting gel plugs with sequencing grade modified porcine trypsin (12.5 ng/L, Promega, Madison, WI). The peptides were extracted, dried in a vacuum, centrifuged, and redissolved in 10 to 20 µL of 0.1% trifluoroacetic acid.

For Nano electrospray MS/MS identification, mass spectrometric measurements were done on a LCQTM Deca XP Plus ion trap mass spectrometer (ThermoFinnigan) equipped with a LCQTM nanospray ionization source. Digested peptides were separated using an Ettan MDLC chromatographic system (GE Healthcare) piloted by Unicorn 5.01 software (GE Healthcare). Three dependent MS/MS spectra of the three most intense peaks were collected following one full scan mass spectrum. Extracted MS/MS spectra were automatically assigned by the Mascot software to the best matching peptide sequence in the NCBI non redundant Database. The identifications were checked by using various peptides obtained by digestion.
